# Tetrodotoxin and Its Analogues in the Pufferfish *Arothron hispidus* and *A. nigropunctatus* from the Solomon Islands: A Comparison of Their Toxin Profiles with the Same Species from Okinawa, Japan

**DOI:** 10.3390/toxins7093436

**Published:** 2015-08-26

**Authors:** Clyde Gorapava Puilingi, Yuta Kudo, Yuko Cho, Keiichi Konoki, Mari Yotsu-Yamashita

**Affiliations:** Graduate School of Agricultural Science, Tohoku University, 1-1 Tsutsumidori-Amamiyamachi, Aoba-ku, Sendai, Miyagi 981-8555, Japan; E-Mails: clyde.puilingi@yahoo.com (C.G.P.); yuta.kudo.p7@dc.tohoku.ac.jp (Y.K.); choyuko@m.tohoku.ac.jp (Y.C.); konoki@m.tohoku.ac.jp (K.K.)

**Keywords:** tetrodotoxin, saxitoxin, *Arothron*, pufferfish, Solomon Islands

## Abstract

Pufferfish poisoning has not been well documented in the South Pacific, although fish and other seafood are sources of protein in these island nations. In this study, tetrodotoxin (TTX) and its analogues in each organ of the pufferfish *Arothron hispidus* and *A. nigropunctatus* collected in the Solomon Islands were investigated using high resolution LC-MS. The toxin profiles of the same two species of pufferfish from Okinawa, Japan were also examined for comparison. TTXs concentrations were higher in the skin of both species from both regions, and relatively lower in the liver, ovary, testis, stomach, intestine, and flesh. Due to higher TTX concentrations (51.0 and 28.7 µg/g at highest) detected in the skin of the two species from the Solomon Islands (saxitoxin was <0.02 µg/g), these species should be banned from consumption. Similar results were obtained from fish collected in Okinawa, Japan: TTX in the skin of *A. hispidus* and *A. nigropunctatus* were 12.7 and 255 µg/g, respectively, at highest, and saxitoxin was also detected in the skin (2.80 µg/g at highest) and ovary of *A. hispidus*. TTX, 5,6,11-trideoxyTTX (with its 4-*epi* form), and its anhydro forms were the most abundant, and 11-oxoTTX was commonly detected in the skin.

## 1. Introduction

**Figure 1:**
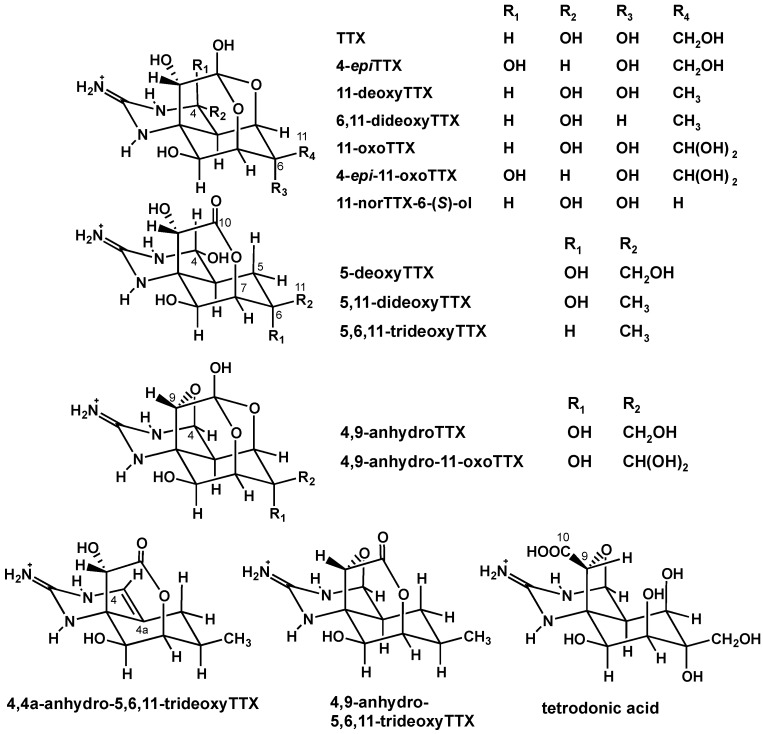
The structures of TTX and its analogues.

Tetrodotoxin (TTX) is a well-studied neurotoxin known for its distribution in pufferfish [1] and marine invertebrates such as snails [2], crabs [3], starfish [4], blue-ringed octopus [5], and sea slugs [6]. Recently, TTX was also detected in bivalve mollusks: New Zealand clams [7], mussels and oysters in England [8], and also in Greek shellfish [9]. Generally, TTX blocks the voltage-gated sodium ion channels, incapacitating nerve conduction and muscle action potentials, causing progressive paralysis and death due to failure of the respiratory system [10,11]. TTX, as the primary agent of pufferfish poisoning, is reported to be produced by marine bacteria and accumulated in the pufferfish via the food chain [12,13]. TTX in newts was reported to be an excellent defense strategy to ward off predators [14], and TTX is used to capture mobile prey by flatworms in Guam [15]. We have found various TTX analogues (Figure 1) which can be further classified into (1) hemilactal type analogues, (2) 5-deoxy-10,7-lactone type analogues, (3) 4,9- and 4,4a-anhydro type analogues and (4) tetrodonic acid type analogue [16,17]. Based on the structures of these analogues, we predicted a stepwise oxidative pathway of TTX in marine animals [17]. Additionally, we recently found C5–C10 directly bonded TTX analogues in newts, suggesting a monoterpene origin of TTX [18].

Toxicity experiments using mouse bioassays have established an LD_50_ (50% lethal dose, mice, i.p.) of TTX and few analogues. For instance, the LD_50_ of TTX [10], 11-deoxyTTX [19] and 6,11-dideoxyTTX [20] were 10 μg/kg, 70 μg/kg, and 420 μg/kg, respectively, while for 5,6,11-trideoxyTTX, LD_99_ was 750 μg/kg [21]. The toxicity of 11-oxoTTX was first reported as 120 µg/kg (minimum lethal dose, mice, i.p.) [22], but then re-examined on rat skeletal muscle fibers with an ED_50_ of 0.7 nM compared to TTX 4.1 nM [23], suggesting that 11-oxoTTX is more toxic than TTX. Furthermore, higher activity was also reflected in the smaller *K*_d_ values of 11-oxoTTX (to rat brain membrane) than TTX [24].

Past incidences of pufferfish food poisoning were reported in several countries including Thailand [25], Bangladesh [26], Hong Kong [27], Singapore [28], Japan [29], Suez Canal region [30], Eastern Mediterranean Sea [31], and several cases in the United States originating mainly from Asian dried pufferfish believed to be illegally imported [32,33]. Reports on these global occurrences and its potential expansion indicate that proper research on the specific toxic species and their distribution is of great importance. However, unlike ciguatera and other seafood poisoning, pufferfish poisoning is not well documented in the South Pacific region although several incidences matching its description have been reported in localized rural areas [34]. The Solomon Islands in particular does not have clear records of victims suffering from pufferfish poisoning. Past undocumented incidents may have triggered a precautionary knowledge passed down through successive generations of the potential dangers of pufferfish consumption. However, because fish and other seafood are a source of protein to the citizens of this island nation, and together with socioeconomic pressures that may affect food security and possibly alter food selectivity, we believe this investigation is imperative as well as informative to the citizens regarding potential risk of pufferfish poisoning. 

Besides TTX, some pufferfish also accumulate paralytic shellfish toxins. The presence of saxitoxin (STX) and decarbamoyl STX (dcSTX) has been reported in pufferfish collected in United States [35], South-eastern Asia, and Japan [36,37,38]. 

In this study, the pufferfish *Arothron hispidus* and *A. nigropunctatus* collected in the Solomon Islands were investigated for TTX and its analogues, as well as STXs for the first time, using high resolution hydrophilic interaction chromatography (HR-HILIC) LC-MS. The toxin profiles of the same two species from Okinawa, Japan, were also examined and compared with those from the Solomon Islands. In addition, two specimens of *Diodon holocanthus* from the Solomon Islands, were also tested for the presence of TTXs, because the skin, flesh, and testis of *D. holocanthus* are officially allowed for consumption in Japan [39].

## 2. Results

### 2.1. Analysis of TTX and Its Analogues in the Pufferfish from the Solomon Islands

#### 2.1.1. Preliminary Analysis of TTX and Its Analogues in the Skin of *A. hispidus* from the Solomon Islands using LC-FLD

**Figure 2:**
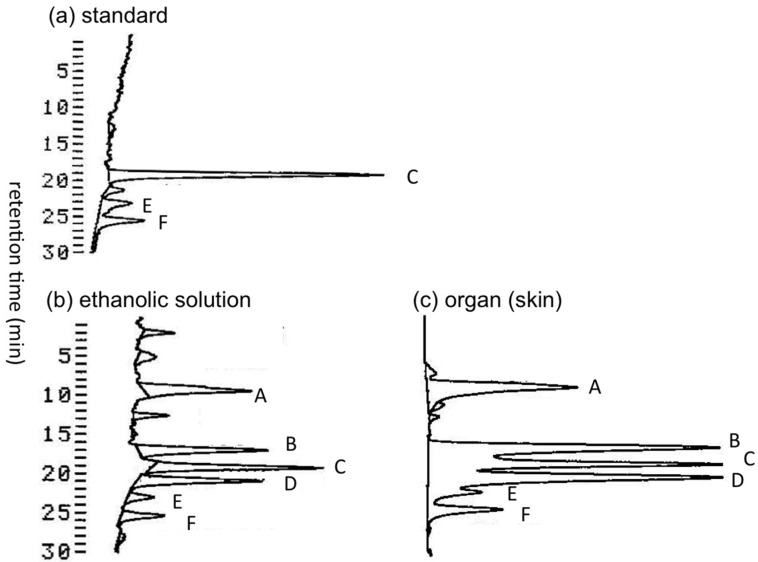
The LC-FLD chromatograms of the skin extracts of the *A. hispidus* No.3 specimen from the Solomon Islands. (**a**) The TTXs standard solution (5 µL) containing TTX 1.0 µg/mL, 4-*epi*TTX 0.18 µg/mL, and 4,9-anhydroTTX 0.24 µg/mL (attenuation 3), (**b**) Aliquot sample (6 µL) prepared from the ethanol-water (7:3, *v/v*) solution used to soak the skin of *A. hispidus* No.3 specimen (10.5 mL/g) (attenuation 6), (**c**) The sample solution (2 µL) prepared from 0.2 M acetic acid (*v/v*) extract of the skin of *A. hispidus* No.3 specimen soaked in ethanolic solution (5.0 mL/g) (attenuation 6) (see Section 4.3). A: tetrodonic acid types, B: 11-oxoTTX and 4-*epi*-11-oxoTTX, C: TTX, D: 4,9-anhydro-11-oxoTTX, E: 4-*epi*TTX, F: 4,9-anhydroTTX. Chromatographic condition: Develosil C30 UG-5 (0.46 x25 cm) column; 30 mM ammonium heptafluorobutyrate buffer (pH 5.0) and 10 mM ammonium formate buffer (pH 5.0) containing 1% (*v/v*) acetonitrile as the solvent, flow rate 0.4 mL/min (see Section 4.5).

Transportation of pufferfish samples from Solomon Islands to Japan under frozen condition was not possible. Therefore, immediately after collection, the pufferfish specimens were dissected into organs and soaked in ethanol-water (7:3, *v/v*) for shipping to Tohoku University (Sendai, Japan) at room temperature. We first examined whether TTXs in the pufferfish samples were decomposed during the transportation period of approximately two weeks. Under such conditions, certain TTX and its analogues might be hydrolyzed to tetrodonic acid (Figure 1) type compounds [16,40], which are detectable using liquid chromatography-fluorescent detection (LC-FLD) for TTXs [41,42] without specific pre-purification. TTXs in the ethanolic solution used to soak the skin of *A. hispidus* (three specimens), and in the skin soaked in this ethanolic solution were both analyzed by LC-FLD. The LC-FLD chromatograms thus obtained in the skin of the *A. hispidus* No.3 specimen are shown in Figure 2. The peak area of tetrodonic acid type analogues (peak A) was approximately 2/3 of TTX (peak C) in both the ethanolic solution (Figure 2b) and the extract of the skin (Figure 2c). The peaks of 11-oxoTTX and 4-*epi*-11-oxoTTX (peak B, not separated), 4,9-anhydro-11-oxoTTX (peak D), 4-*epi*TTX (peak E) and 4,9-anhydroTTX (peak F) are clearly observed. These data suggest that the pufferfish samples transported from the Solomon Islands are still suitable for analysis of TTXs, even though TTXs were partially transformed to tetrodonic acid type analogues. 

**Figure 3:**
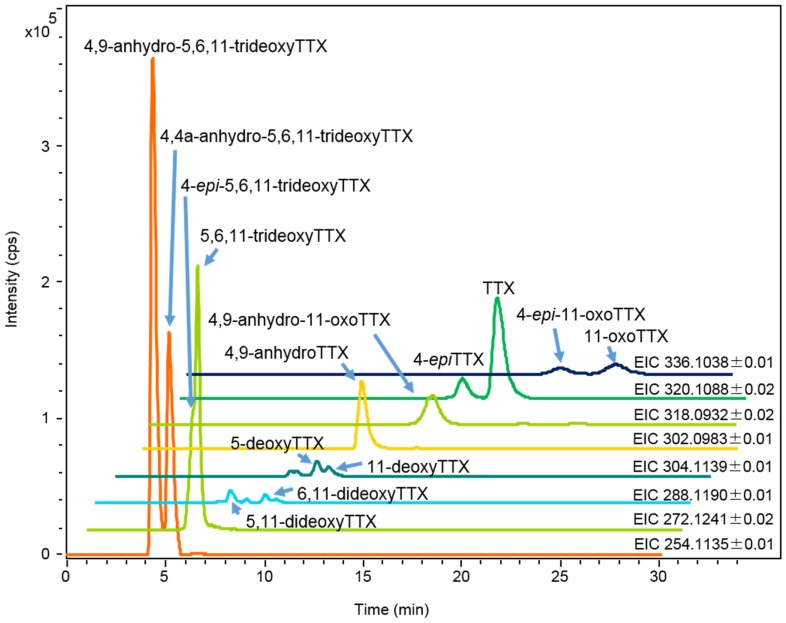
HR-LC-MS chromatograms (extracted ion chromatograms: EICs) of charcoal treated sample solution (2 µL) prepared from the skin of *A. hispidus* No.3 specimen (25 mL/g) from the Solomon Islands soaked in ethanolic solution. The sample solution contained TTX 1.4 µg/mL, 4-*epi*TTX 0.30 µg/mL, 4,9-anhydroTTX 0.80 µg/mL, 5-deoxyTTX 0.13 µg/mL, 6,11-dideoxyTTX 0.03 µg/mL, 5,11-dideoxyTTX 0.09 µg/mL, 5,6,11-trideoxyTTX and 4-*epi*-5,6,11-trideoxyTTX (total of two analogues 1.8 µg/mL), 4,4a-anhydro-5,6,11-trideoxyTTX 2.7 µg/mL, 11-oxoTTX 0.50 µg/mL, 4-*epi*-11-oxoTTX 0.50 µg/mL, and 4,9-anhydro-11-oxoTTX 0.60 µg/mL.

#### 2.1.2. Characterization and Quantitation of TTX and Its Analogues Using High Resolution-LC-MS

**Figure 4:**
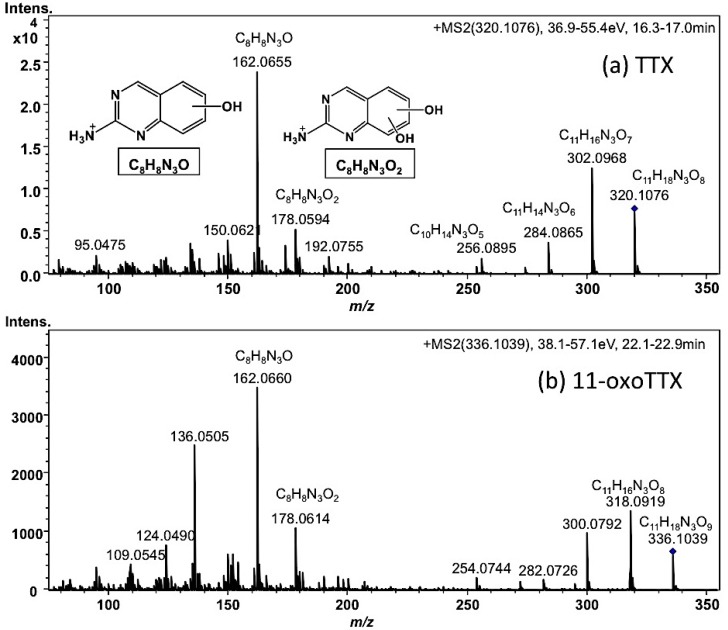
HR-LC-MS/MS spectra of TTX (a) and 11-oxoTTX (b) in the skin of *A. hispidus* No.3 specimen from the Solomon Islands. The sample solution (2 µL) was same as shown in Figure 3. The predicted structures of the major fragment ions were superimposed in the spectra.

Each tissue soaked in ethanolic solution was extracted with hot 0.2 M acetic acid (*v/v*). The extract was purified with activated charcoal and analyzed by High Resolution (HR) HILIC-LC-MS (Q-TOF MS) as previously reported [17,43,44,45]. The typical mass chromatograms of major TTX analogues in the skin of *A. hispidus* No.3 specimen are shown in Figure 3. All TTX analogues were quantified by HR-LC-MS based on the standard curve for TTX. However, in the samples from the Solomon Islands, the peak areas of 11-oxoTTX and 4-*epi*-11-oxoTTX on the extracted ion chromatograms (EIC) at *m/z* 336.1014 were smaller than that predicted by the LC-FLD analysis. Probably ionization was suppressed by certain compounds eluted at the same retention time. Therefore, 11-oxoTTX and 4-*epi*-11-oxoTTX were quantified using LC-FLD. The limit of detection (LOD) of all TTX analogues, except 11-oxoTTX and 4-*epi*-11-oxoTTX in the samples from the Solomon Islands, was (*S/N* > 3, 0.01 µg/g), while the limit of quantitation (LOQ) was (*S/N* > 10, 0.03 µg/g). The LOD and LOQ of 11-oxoTTX and 4-*epi*-11-oxoTTX in the samples from the Solomon Islands were 0.04 µg/g and 0.14 µg/g, respectively. TTX, 4,9-anhydroTTX, 5,6,11-trideoxyTTX (with its 4-*epi* form), 4,4a-anhydro-5,6,11-trideoxyTTX [46], and 4,9-anhydro-5,6,11-trideoxyTTX were major TTX analogues in almost all the skin samples. In addition, 11-oxoTTX which is considered more potent than TTX by some authors [23,24], was observed with its 4-*epi* and 4,9-anhydro forms. The MS/MS spectrum of 11-oxoTTX showed a similar fragmentation pattern to that of TTX, detecting *m/z* 162.0660 (C_8_H_8_N_3_O) and 178.0614 (C_8_H_8_N_3_O_2_) ions, corresponding to 2-aminohydroquinazoline and 2-aminodihydroquinazone, respectively [17], as the major fragment ions (Figure 4). Regarding minor analogues, monodeoxyTTX (11-deoxyTTX and 5-deoxyTTX) and dideoxyTTX (5,11-dideoxyTTX and 6,11-dideoxyTTX) were also detected.

#### 2.1.3. Estimation of the Concentrations of TTX and Its Analogues in Each Organ of *A. hispidus*, *A. nigropunctatus*, and *D. holocanthus* from the Solomon Islands

For three specimens of *A. hispidus* and *A. nigropunctatus*, and two specimens of *D. holocanthus* from the Solomon Islands, TTX and its analogues both in the ethanolic solutions and in the soaked organs were separately quantified using HR-LC-MS after prepurification. The results were combined together, according to solvent quantity and tissue weight, to estimate the real concentrations of TTXs in the organs. 5,6,11-TrideoxyTTX and 4,9-anhydro-5,6,11-trideoxyTTX were present relatively higher in the ethanolic solutions compared with the organs. The results of *A. hispidus* and *A. nigropunctatus* (three specimens for each species) from the Solomon Islands are summarized in Table 1 and Table 2, respectively. TTX, 5,6,11-trideoxyTTX, 4,4a-anhydro-5,6,11-trideoxyTTX, and 4,9-anhydro-5,6,11-trideoxyTTX were the most abundant analogues in all organs in these pufferfish species, and 11-oxoTTX which is considered a more potent analogue than TTX [23,24] was detected in the skin of all specimens. TTX concentrations in the skin were generally higher in all specimens, and relatively lower in the liver, ovary, testis, stomach, and intestine. The highest TTX concentrations in the skin of *A. hispidus* and *A. nigropunctatus* were 51.0 and 28.7 µg/g, respectively. Additionally, we confirmed that TTXs in *D. holocanthus* from the Solomon Islands were less than the limit of detection (LOD; *S/N* > 3, 0.01 µg/g) in all the organs tested (skin, liver, gonad, stomach, intestine, and flesh), even though there were only two specimens (a male and a female). 

**Table 1:** The concentrations of TTX and its analogues (µg/g) in the organs of *A. hispidus* from the Solomon Islands.

Organ	Skin	Liver	Ovary	Testis	Stomach	Intestine	Flesh
	*n* = 3	*n* = 3	*n* = 1	*n* = 2	*n* = 3	*n* = 2	*n* = 1
**TTX**	7.20–51.0	0.03–7.99	1.89	<LOD, 11.7	0.58–9.45	1.95, 5.45	0.07
**4-*epi*TTX**	1.80–14.8	<LOD-1.29	0.23	<LOD, 1.93	<LOD-1.52	<LOD, 0.27	<LOD
**4,9-anhydroTTX**	3.60–26.0	<LOD-4.00	<LOD	<LOD, 6.63	<LOD-4.93	<LOD, <LOQ(0.02)	<LOD
**11-deoxyTTX**	0.10–4.32	<LOD-0.67	<LOD	<LOD, 1.17	<LOD-0.66	<LOD, 0.06	<LOD
**5-deoxyTTX**	<LOD-5.51	<LOD-0.21	<LOD	<LOD, 0.35	<LOD-0.16	0.10, 0.10	<LOD
**6,11-dideoxyTTX**	<LOD-1.47	<LOD-0.08	<LOD	<LOD	<LOD-0.06	<LOD	<LOD
**5,11-dideoxyTTX**	<LOD-3.23	<LOD-0.14	<LOD	<LOD	<LOD-0.56	<LOD, 0.16	<LOD
**5,6,11-trideoxyTTX**	1.47–84.3	<LOD-16.4	10.1	<LOD, 14.6	<LOD-19.5	5.54, 7.20	0.05
**4,4a-anhydro-5,6,11-trideoxyTTX**	1.36–70.1	<LOD-10.4	6.74	<LOD, 8.99	7.34–7.92	4.93, 5.36	0.11
**4,9-anhydro-5,6,11-trideoxyTTX**	2.06–133.2	0.34–12.2	7.94	<LOD, 9.15	7.71–21.6	5.04, 10.7	0.11
**11-norTTX-6(*S*)-ol**	<LOD-2.23	<LOD-0.51	0.04	<LOD, 1.02	<LOD-0.74	<LOD, 0.07	<LOD
**11-oxoTTX**	0.32–7.07	<LOD-0.35	<LOD	<LOD, 0.41	<LOD-0.31	< LOQ(0.05), 1.68	<LOD
**4-*epi*-11-oxoTTX**	<LOQ(0.06)-3.53	<LOD-<LOQ(0.06)	<LOD	<LOD, <LOQ(0.09)	<LOD-<LOQ(0.13)	<LOD, 0.26	<LOD
**4,9-anhydro-11-oxoTTX**	<LOD-13.7	<LOD-0.18	0.22	<LOD, 0.58	<LOQ(0.08)-0.41	0.21, 1.72	<LOD

For 11-oxoTTX and 4-*epi*-11-oxoTTX: LOD = 0.04 µg/g (*S/N* > 3), LOQ = 0.14 µg/g (*S/N* > 10), for other analogues: LOD = 0.01 μg/g (*S/N* > 3), LOQ = 0.03 μg/g (*S/N* > 10). 5,6,11-TrideoxyTTX was measured as the mixture with its 4-*epi* form.

**Table 2:** The concentrations of TTX and its analogues (µg/g) in the organs of *A. nigropunctatus* from the Solomon Islands.

Organ	Skin	Liver	Ovary	Testis	Stomach	Intestine
	*n* = 3	*n* = 3	*n* = 2	*n* = 1	*n* = 2	*n* = 1
**TTX**	8.51–28.7	<LOD-19.7	1.14, 19.7	<LOD	1.57, 21.9	0.38
**4-*epi*TTX**	1.61–6.68	<LOD-3.11	0.28, 3.16	<LOD	0.41, 3.04	0.05
**4,9-anhydroTTX**	5.15–15.9	<LOD-10.8	0.91, 8.60	<LOD	<LOD, 11.0	0.19
**11-deoxyTTX**	<LOD-0.83	<LOD	<LOD	<LOD	<LOD	<LOD
**5-deoxyTTX**	<LOD-0.14	<LOD	<LOD	<LOD	<LOD	<LOD
**6,11-dideoxyTTX**	<LOD-0.10	<LOD	<LOD	<LOD	<LOD	<LOD
**5,11-dideoxyTTX**	<LOD	<LOD	<LOD	<LOD	<LOD	<LOD
**5,6,11-trideoxyTTX**	3.99–13.0	<LOD-10.7	1.12, 6.14	<LOD	1.25, 9.17	0.55
**4,4a-anhydro-5,6,11-trideoxyTTX**	2.39–8.44	0.02–10.1	0.92, 5.89	<LOD	1.32, 8.63	0.53
**4,9-anhydro-5,6,11-trideoxyTTX**	3.90–11.1	<LOD-11.0	1.07, 6.73	<LOD	1.31, 8.94	0.54
**11-norTTX-6(*S*)-ol**	0.10–0.84	<LOD	<LOD	<LOD	<LOD	<LOD
**11-oxoTTX**	0.16–0.67	<LOD-0.77	<LOD, 0.56	<LOD	<LOQ(0.05), 0.80	<LOD
**4-*epi*-11-oxoTTX**	<LOQ(0.09)-0.50	<LOD-0.27	<LOD, 0.22	<LOD	<LOD, 0.26	<LOD
**4,9-anhydro-11-oxo-TTX**	0.33–1.49	<LOD-1.11	<LOD, 0.66	<LOD	0.19, 0.93	<LOQ(0.07)

For 11-oxoTTX and 4-*epi*-11-oxoTTX: LOD = 0.04 µg/g (*S/N* > 3), LOQ =0.14 µg/g (*S/N* > 10), for other analogues: LOD = 0.01 μg/g (*S/N* > 3), LOQ = 0.03 μg/g (*S/N* > 10). 5,6,11-TrideoxyTTX was measured as the mixture with its 4-*epi* form.

### 2.2. The concentrations of TTX and Its Analogues in Each Organ of A. hispidus and A. nigropunctatus From Okinawa, Japan 

The pufferfish specimens from Okinawa were transported live to Sendai and immediately frozen at −25 °C. TTXs in each organ of three specimens of *A. hispidus* and four specimens of *A. nigropunctatus* were extracted and pre-purified with activated charcoal, and were analyzed using HR-LC-MS. The data obtained for *A. hispidus* and *A. nigropunctatus* are shown in Table 3 and Table 4, respectively. The mass chromatograms and organ distribution of toxins in these *Arothron* species from Okinawa, Japan, were very similar to those from the Solomon Islands. However, one specimen of *A. nigropunctatus* from Okinawa (No.1 male specimen) showed very high levels of TTX and 11-oxoTTX in the skin, 255 and 42.4 µg/g, respectively. TTX concentration in the stomach of this specimen (25.5 µg/g) was higher than other organs. 11-OxoTTX was detected in the skin of all specimens of both species, same as those from the Solomon Islands.

**Table 3:** The concentrations of TTX and its analogues (µg/g) in the organs of *A. hispidus* from Okinawa, Japan.

Organ	Skin	Liver	Ovary	Testis	Stomach	Intestine	Flesh	Spleen
	*n* = 3	*n* = 3	*n* = 1	*n* = 2	*n* = 3	*n* = 3	*n* = 3	*n* = 3
**TTX**	4.26–12.7	0.17–0.55	0.37	1.08, 1.11	<LOD-2.44	1.61-2.05	0.23–0.61	0.15–2.09
**4-*epi*TTX**	0.40-2.75	<LOD-0.03	0.04	0.07, 0.17	<LOD-0.52	0.10-0.36	<LOD-0.04	<LOD-0.25
**4,9-anhydroTTX**	2.22–8.84	<LOD-0.26	0.17	0.46, 0.52	<LOD-1.74	0.59–1.36	0.04–0.18	<LOD-0.74
**11-deoxyTTX**	1.34–2.12	<LOD	<LOD	0.04, 0.18	<LOD-0.21	<LOD-0.32	<LOD	<LOD-0.11
**5-deoxyTTX**	0.73–1.19	<LOD	<LOD	0.11, 0.16	<LOD-0.05	<LOD-0.31	<LOD	<LOD
**6,11-dideoxyTTX**	0.12–0.31	<LOD	<LOD	<LOD, 0.05	<LOD	<LOD-<LOQ(0.02)	<LOD	<LOD
**5,11-dideoxyTTX**	0.11–0.52	<LOD	<LOD	<LOD, 0.03	<LOD	<LOD-<LOQ(0.02)	<LOD	<LOD
**5,6,11-trideoxyTTX**	1.09–6.02	< 0.06	0.18	< 0.20, 0.56	< 0.10-0.19	0.21–0.36	0.03–0.23	<LOD-0.10
**4,4a-anhydro-5,6,11-trideoxyTTX**	1.91–18.6	0.31–0.46	0.33	0.24, 0.67	< 0.14–0.80	0.42–0.73	0.07–0.31	0.03–0.55
**4,9-anhydro-5,6,11-trideoxyTTX**	7.36–46.7	0.44–1.66	1.11	1.70, 3.79	< 0.46–2.23	1.77–2.80	0.21–0.45	0.15–1.10
**11-norTTX-6(*S*)-ol**	0.27–0.92	<LOD	<LOD	0.04, 0.06	<LOD-0.18	0.08–0.12	<LOD-0.05	<LOD-0.12
**11-oxoTTX**	<LOQ(0.07)-0.20	<LOD	<LOD	<LOD, <LOQ(0.12)	<LOD	<LOD- <LOQ(0.05)	<LOD	<LOD
**4-*epi*-11-oxoTTX**	<LOD	<LOD	<LOD	<LOD	<LOD	<LOD	<LOD	<LOD
**4,9-anhydro-11-oxoTTX**	<LOQ(0.12)-0.15	<LOD	<LOD	<LOD, <LOQ(0.07)	<LOD-< LOQ(0.08)	<LOD- <LOQ(0.09)	<LOD	<LOD

For 11-oxoTTX, 4-*epi*-11-oxoTTX and 4,9-anhyydro-11-oxoTTX: LOD = 0.04 µg/g (*S/N* > 3), LOQ = 0.14 µg/g (*S/N* > 10), for other analogues: LOD = 0.01 μg/g (*S/N* > 3), LOQ = 0.03 μg/g (*S/N* > 10). 5,6,11-TrideoxyTTX was measured as the mixture with its 4-*epi* form.

**Table 4:** The concentrations of TTX and its analogues (µg/g) in the organs of *A. nigropunctatus* from Okinawa, Japan.

Organ	Skin	Liver	Ovary	Testis	Stomach	Intestine	Flesh	Spleen
	*n* = 4	*n* = 4	*n* = 1	*n* = 3	*n* = 4	*n* = 4	*n* = 4	*n* = 4
**TTX**	12.6–255	0.29–7.46	1.02	0.26–10.1	0.57–25.5	1.22–4.51	0.19–4.26	0.85–4.19
**4-*epi*TTX**	1.19–27.9	<LOD-0.49	0.09	<LOD-2.64	0.07–2.05	0.07–0.35	<LOD-0.92	0.18–0.62
**4,9-anhydroTTX**	5.39–96.6	<LOD-2.75	0.50	0.13–6.13	0.24–9.65	0.52–2.41	<LOD-1.82	0.85–2.26
**11-deoxyTTX**	1.25–14.9	<LOD-0.54	0.10	0.02–3.13	0.06–2.19	0.15–0.65	<LOD-0.88	0.10–0.76
**5-deoxyTTX**	<LOD-4.03	<LOD-0.23	0.09	0.10–3.80	<LOD-5.41	<LOD-0.54	<LOD-0.62	<LOD-0.80
**6,11-dideoxyTTX**	0.40–1.77	<LOD-0.07	0.05	<LOD-1.31	0.02–0.84	<LOD-0.27	<LOD	<LOD-0.43
**5,11-dideoxyTTX**	0.52–2.92	<LOD-0.06	<LOQ(0.02)	0.10–0.94	<LOD-1.03	<LOD-0.12	<LOD-0.18	<LOD-0.14
**5,6,11-trideoxyTTX**	15.3–275	<LOD -3.69	0.39	0.61–22.3	0.28–25.2	<LOD-8.40	0.04–3.92	0.74–10.0
**4,4a-anhydro-5,6,11-tri-deoxyTTX**	6.81–178	0.34–2.69	0.56	0.45–15.3	0.39–10.2	0.74–5.31	0.34–3.32	1.01–5.69
**4,9-anhydro-5,6,11-trideoxyTTX**	43–438	0.30–8.28	3.48	2.05–26.9	1.42–77.0	0.93–11.0	0.70–4.79	3.74–10.5
**11-norTTX-6(*S*)-ol**	0.11–9.72	<LOD-0.30	<LOD	<LOD-0.32	<LOD-0.11	<LOD-0.19	<LOD-0.11	<LOD-0.15
**11-oxoTTX**	3.94–42.4	<LOQ(0.05)-0.84	<LOQ(0.12)	<LOQ(0.07)-0.82	<LOQ(0.05)-9.98	<LOQ(0.12)-0.53	<LOD-<LOQ(0.11)	0.12–0.46
**4-*epi-*11-oxoTTX**	0.51–6.54	<LOD-0.16	<LOD	<LOD-0.16	<LOD-1.81	<LOD-<LOQ(0.10)	<LOD	0.05–0.16
**4,9-anhydro-11-oxoTTX**	2.57–25.0	<LOQ(0.04)-0.5	0.15	< LOQ(0.05)-0.69	<LOQ(0.11)-4.15	0.20–0.41	<LOD-0.35	0.09–0.53

For 11-oxoTTX, 4-*epi*-11-oxoTTX and 4,9-anhydro-11-oxoTTX: LOD = 0.04 µg/g (*S/N* > 3), LOQ = 0.14 µg/g (*S/N* > 10), for other analogues: LOD = 0.01 μg/g (*S/N* > 3), LOQ = 0.03 μg/g (*S/N* > 10). 5,6,11-TrideoxyTTX was measured as the mixture with its 4-*epi* form.

### 2.3. Analysis of Saxitoxin (STX) and Its Analogues in A. hispidus and A. nigropunctatus from the Solomon Islands and Okinawa, Japan

The presence of STX, neoSTX, and dcSTX was investigated in the skin and ovary of the pufferfish from the Solomon Islands and Okinawa, Japan, using HR-LC-MS. The concentration of STX was below the LOD (0.02 µg/g) in the skin and ovary of all pufferfish from the Solomon Islands and all four specimens of *A. nigropunctatus* from Okinawa, Japan. However, STX was detected in the skin (0.09, 2.80, 0.34 µg/g) of all three specimens of *A. hispidus* from Okinawa, Japan. A female specimen *A. hispidus* (No. 2) from Okinawa, which possessed highest STX (2.80 µg/g) and dcSTX (0.66 µg/g) in the skin, also contained trace amount of STX (0.04 µg/g: between LOD and LOQ) in the ovary. In other samples, dcSTX was not detected. NeoSTX was not detected in all samples. LOD (*S/N* > 3) and LOQ (*S/N* > 10) for STX were 0.02 µg/g and 0.07 µg/g, neoSTX, 0.05 µg/g and 0.18 µg/g, and dcSTX, 0.07 µg/g and 0.25 µg/g, respectively.

## 3. Discussion

We estimated the concentrations of TTX and its analogues in the pufferfish, *A. hispidus* and *A. nigropunctatus*, from the Solomon Islands and Okinawa, Japan, using HR-LC-MS. The pufferfish organs from the Solomon Islands were transported in ethanol-water (7:3, *v/v*) at room temperature to Japan. Based on the LC-FLD analysis, the amounts of tetrodonic acid type analogs, likely derived from TTXs under such condition, was estimated to be almost 2/3 of total TTX. Thus, we should note that the amount of TTX in the samples from the Solomon Islands (Table 1 and Table 2) could be underestimated. However, we believe that the data obtained in this study must be reported to warn of the high toxicity of these species in the Solomon Islands. The flesh of only one specimen of *A. hispidus* from the Solomon Islands was analyzed, due to limited number storage tubes and volume of ethanolic solution available during pufferfish collection. TTX in that flesh was low (0.07 µg/g), however, the levels of TTX in the skin of *A. hispidus* and *A. nigropunctatus* were high in all specimens of both species. In addition, TTX was detected in the flesh of all specimens of these species from Okinawa, Japan (*A. hispidus* from 0.23 to 0.61 µg/g; *A. nigropunctatus* from 0.19 to 4.26 µg/g). Based on these data, these species, including flesh, from the Solomon Islands and Okinawa, Japan, should be banned from consumption. Khora *et al.* [47] and Teruya *et al*. [48] also indicated high toxicity in the flesh of these *Arothron* species from Okinawa, Japan, based on the result of a mouse bioassay. 

The distribution of TTXs in the organs of *A. hispidus* and *A. nigropunctatus* from the Solomon Islands and Okinawa, Japan, revealed that the skin is the major location of TTX accumulation. The skin of *A. hispidus* and *A. nigropunctatus* from the Solomon Islands contained 7.20 to 51.0 µg/g and 8.51 to 28.7 µg/g, respectively, while those from Okinawa, Japan, were 4.26 to 12.7 µg/g (*A. hispidus*), and 12.6 to 255 µg/g (*A. nigropunctatus*). These data suggested that the concentration of TTX is highly variable among specimens. Our findings agree with previous reports that showed higher toxicity to mice in the skin tissues of the same species collected in Okinawa area, Japan [47]. A study on the Hawaiian *A. hispidus* suggested high levels of TTX producing bacteria isolated from the skin [49], although the details have not yet been clarified. The levels of TTX in the liver and gonads are usually high in common Japanese pufferfish species in genus *Takifugu* (*Fugu*) [50], but they were low in the *Arothron* species compared with those of the skin. We assume that different regions, with their different biotic and abiotic factors, may play an important role in the TTX accumulation ability of pufferfish. More specimens are needed to compare the TTX distribution profile in the organs of the pufferfish from the Pacific region. The difference of toxin distribution pattern between *Takifugu* and *Arothron* is probably caused by the difference of some proteins which are implicated in TTX accumulation system. We previously found a glycoprotein, named as PSTBP (puffer fish saxitoxin and tetrodotoxin binding protein), that binds to TTX and STX in the blood plasma of *Takifugu pardalis* [51], and also, it was reported that PSTBP was commonly detected in several *Takifugu* species [52,53]. We are planning to examine the presence of PSTBP-like protein in *Arothron* species to get a hint that will help explain the difference of TTX-accumulation tissues between *Takifugu* and *Arothron* genus.

We also analyzed TTX and its analogues in several organs of two specimens of *D. holocanthus* from the Solomon Islands, and confirmed that the concentrations of all TTX analogues were below the LOD (*S/N* > 3, 0.01 µg/g). We will further analyze more specimens for confirmation in the future. In Japan, the skin, flesh, and testis of *D. holocanthus* collected in Japan are officially allowed for consumption [39].

As previously noted [40,50], 4,9-anhydroTTX is one of the major TTX analogues in almost all toxic pufferfish tissues, because it is chemically equilibrated with TTX. Likewise, 5,6,11-trideoxyTTX, 4,4a-anhydro-5,6,11-trideoxyTTX and 4,9-anhydro-5,6,11-trideoxyTTX also showed significant concentrations in all toxic pufferfish being investigated. For instance, 4,9-anhydro-5,6,11-trideoxyTTX concentrations even surpassed those of TTX in tissues of a few individuals (Table 1, Table 3 and Table 4). Previous studies revealed that 5,6,11-trideoxyTTX, which is also a major analogue in other pufferfish species such as Japanese *Takifugu pardalis* [50] and European trumpet shell *Charonia lampas* [54], has a relatively low toxicity (LD_99_ 720 µg/kg, mice, i.p.) [21] compared to TTX and other toxic analogues. In addition, 11-oxoTTX was commonly detected in *A. nigropunctatus* and *A. hispidus* from the Solomon Islands and Okinawa, Japan (Table 1, Table 2, Table 3 and Table 4). This analogue was originally isolated from Micronesian *A. nigropunctatus* [22], however, this is the first identification in *A. hispidus*. 11-OxoTTX has been also found in the Brazialian frog [55], the red spotted newt [56], the blue-ringed octopus [57], the marine snail [58] and the xanthid crab [59]. Because 11-oxoTTX is considered as more potent than TTX by some authors [23,24], its presence in *A. hispidus* and *A. nigropunctatus* poses a potential risk of pufferfish poisoning. 

The presence of STX and its analogues were also tested in the skin and ovary of *A. hispidus* and *A. nigropunctatus* from the Solomon Islands and Okinawa, Japan. STX was detected in the skin of all three specimens of *A. hispidus* from Okinawa (0.09, 2.80, 0.34 µg/g), as well as in the ovary of one specimen, but not detected in the pufferfish from the Solomon Islands (< 0.02 µg/g). It is notable that the concentration of STX equivalents (STX + dcSTX) was 3.46 µg/g in the skin of an *A. hispidus* specimen from Okinawa. This is higher than the global regulatory limit of 800 μg of STX equivalents per kg of shellfish meat (e.g. Codex, EU, US, Japan) [60,61,62,63]. Analysis of STX in more specimens from both regions is needed, because Nakashima *et al.* [36] reported that STX and dcSTX were detected in the ovary of *Arothron firmamentum* collected in Japan at high concentration. From the results obtained in this present study, we believe this investigation is informative and also important for the people of Solomon Islands regarding potential risk of pufferfish poisoning. 

## 4. Experimental Section

### 4.1. Standards and Reagents

Semi-purified TTXs mixture prepared from the ovary of *Takifugu poecilonolus* using a charcoal column [21] was used as the standard for TTXs as described previously [17]. The authentic 11-oxoTTX was prepared from TTX by chemical oxidation [24]. TTX used to draw the standard curve for HR-LC-MS was highly purified by our group. The standard mixture of STX, dcSTX, and neoSTX were prepared (approved under AOAC guidelines) by Oshima [64]. All of the solvents for LC and the reagents were purchased from Wako Pure Chemical Industries (Osaka, Japan), while ammonium formate for LC-MS, heptafluorobutyrate acid, and acetonitrile for LC-FLD were purchased from Sigma-Aldrich (St. Louis, MO, USA).

### 4.2. Sampling and Test Materials 

Three specimens of *Arothron hispidus*, three specimens of *A. nigropunctatus* and two specimens of *Diodon holocanthus* were collected in the Tomba islets (latitude 8.427° S, longitude 157.929° W) Marovo Lagoon, Solomon Islands, in May and July 2014. The body length, body weight, and gender of the three specimens of *A. hispidus* were No.1 19 cm, 130 g, female; No.2 17 cm, 118 g, male; No.3 18 cm, 120 g, male, those of the three specimens of *A. nigropunctatus* were No.1 19 cm, 163 g, female; No.2 37 cm, 320 g, male; No.3 20 cm, 180 g, female, and those of two specimens of *D. holocanthus* were 30–35 cm, 300–350 g, No.1 female, No.2 male. Immediately after collection, the pufferfish were dissected into respective organs (skin, liver, gonad, stomach, intestine, flesh), and a part or whole of each organ were separately soaked in approximately 15 mL of ethanol-water (7:3, *v/v*) in a disposable plastic tube (TPP, Trasadingen, Switzerland). Samples were transported to Tohoku University, Sendai, Japan, at room temperature, because preserving samples at low temperature (freezing) was not possible due to the lack of proper freezing equipment and the long transit time for air-shipment from the Solomon Islands. Upon arrival samples were kept below −25 °C until use. From Okinawa, Japan, four living specimens of *A. nigropunctatus* (No.1 15 cm, 67 g, male; No.2 21 cm, 375 g, male; No.3 14 cm, 70 g, male; No.4 16 cm, 130 g, female) and three living specimens of *A. hispidus* (No.1 17 cm, 112 g, male; No.2 16.5 cm, 124.4 g, female; No.3 10.0 cm, 31.3 g, male), collected from April to June 2015, were sent to Tohoku University. After arrival, they were dissected into organs and kept frozen at −25 °C until use.

### 4.3. Preliminary Analysis of TTXs in the Skin of A. hispidus from the Solomon Islands Using LC-FLD for TTXs

An aliquot (0.5 mL) of the ethanol-water (7:3, *v/v*) solution used to soak the skin of *A. hispidus* three specimens from the Solomon Islands was acidified by acetic acid and dried *in vacuo*, and then dissolved in 0.5 mL of 0.05 M acetic acid. After centrifugation, a part of the supernatant was diluted ten-fold with 0.05 M acetic acid. An aliquot (2 µL) of this solution was applied to LC-FLD for TTXs measurement. Similarly, the skin from these specimens was removed from the ethanolic solution, and a part (0.25 g) was extracted with 1.25 mL of 0.2 M acetic acid (*v/v*) in boiling water. The extract was centrifuged at 15,000× *g* and an aliquot (2 µL) of the supernatant was applied to LC-FLD [41,42].

### 4.4. Sample Preparation for HR-LC-MS Analysis

#### 4.4.1. From the Ethanolic Solution Used to Soak the Pufferfish Organs from the Solomon Islands

An aliquot (0.5 mL) of ethanol-water (7:3, *v/v*) solution (from a total of 14.9 mL for the skin of *A. hispidus* No.3 for example) was mixed with 5 µL of acetic acid, and dried *in vacuo*. The obtained residue was dissolved in 0.5 mL of 0.05 M acetic acid and neutralized with 1 M NaOH aqueous solution, and then applied to activated charcoal (column volume: 250 μL, for chromatography, Wako, Osaka, Japan) packed in a glass pipette equilibrated with water. After washing charcoal with 0.75 mL of water, TTXs were eluted with 1.5 mL of acetic acid-ethanol-water (2:50:48, *v/v*). An aliquot (50 µL) of this eluate was dried *in vacuo* and dissolved in 50 µL of 0.05 M acetic acid, and then filtered through Cosmospin Filter H (Nacalai Tesque, Inc., Kyoto, Japan). After dilution of the filtrate with 0.05 M acetic acid, an aliquot of this solution was used for HR-LC-MS. The recovery of TTXs in the organs from the activated charcoal was estimated at approximately 70% using LC-FLD. Therefore, the final TTXs concentrations in the organs were recalculated based on this recovery ratio.

#### 4.4.2. From the Organs of Solomon Islands Pufferfish Soaked in Ethanolic Solution, and from the Frozen Organs of Pufferfish from Okinawa, Japan

The organ was homogenized and an aliquot (0.25 g) was mixed with 1.25 mL of acetic acid-water (1:99, *v/v*). The mixture was boiled for 10 minutes in a 1.5 mL micro tube, and centrifuged at 15,000× *g* for 15 minutes at 4 °C. Half of the supernatant was neutralized with 1 M NaOH, and then applied to the charcoal (column volume 250 µL). The sample was treated by the same procedure as described above Section (4.4.1).

### 4.5. LC-FLD for TTXs

Post-column LC-fluorescent detection (LC-FLD) for TTXs was performed as described previously [41,42]. Briefly, The LC condition was as follow: Develosil C30 UG-5 (0.46 cm i.d. × 25 cm) (Nomura Chemical, Seto, Japan) with 30 mM ammonium heptafluorobutyrate buffer (pH 5.0) and 10 mM ammonium formate buffer (pH 5.0) containing 1% (*v/v*) acetonitrile at a flow rate of 0.4 mL/min; for the post column reaction 4 M NaOH at a flow rate of 0.7 mL/min to be heated at 105 °C in the stainless tube (0.46 mm i.d. × 3.5 m). The chromatography was performed at 20 °C. The derived fluorophores by post column reaction were detected by a Jasco FP2025 plus fluoromonitor (Jasco, Tokyo, Japan) setting excitation wavelength at 365 nm and emission wavelength at 510 nm.

### 4.6. High Resolution LC-MS and LC-MS/MS for TTXs

HILIC LC-MS and MS/MS methods were performed as previously reported [17]. Briefly, a Shimadzu Nexera UHPLC System (Shimadzu, Kyoto, Japan) was used as the liquid chromatography system, and consisted of a LC-10AD pump (Shimadzu, Kyoto, Japan), autosampler (SIL-30AC, Shimadzu) and a TSKgel Amide-80 column (150 × 2.0 mm i.d., particle size 5 µm, Tosoh, Tokyo, Japan). The mobile phase, 16 mM ammonium formate in water/acetonitrile/formic acid (30:70:0.002, *v/v*) has a flow rate of 0.2 mL/min at 28 °C in the isocratic mode with an injection volume of 2–3 μL and run time of 25 min. The liquid chromatography system was connected to a Q-TOF MS spectrometer, MicrOTOFQII (Bruker Daltonics, Bremen, Germany), equipped with an ESI source. The conditions of the MS spectrometer were as follows: positive ionization mode, dry gas: nitrogen 7 L/min, dry temperature: 180 °C, nebulizer: 1.6 Bar, capillary: −4500 V. The ions at *m/z* 320.1088 (TTX, 4-*epi*TTX), 302.0983 (4,9-anhydroTTX), 304.1139 (11-deoxyTTX, 5-deoxyTTX), 288.1190 (6,11-trideoxyTTX, 5,11-dideoxyTTX), 272.1241 (5,6,11-trideoxyTTX, 4-*epi*-5,6,11-trideoxyTTX) and 254.1135 (4,9-anhydro-5,6,11-trideoxyTTX, 4,4a-anhydro-5,6,11-trideoxyTTX), 336.1038 (11-oxoTTX, 4-*epi*-11-oxoTTX), 318.0932 (4,9-anhydro-11-oxoTTX) corresponding to the [*M* + *H*]^+^ ions were analyzed in the extracted ion chromatograms (EIC). The mass tolerance width for the ions are as shown in Figure 3 (0.01 or 0.02). As described in the Section 2.1.2, all TTX analogues were quantified by HR-LC/MS based on the standard curve for TTX. However, in the samples from the Solomon Islands, the peak of 11-oxoTTX and 4-*epi*-11-oxoTTX were suppressed by certain compounds eluted at the same retention time. Therefore, in that case, 11-oxoTTX and 4-*epi*-11-oxoTTX were quantified using LC-FLD. The limit of detection (LOD) of all TTX analogues except 11-oxoTTX and 4-*epi*-11-oxoTTX were (*S/N* > 3, 0.01 µg/g) and the limit of quantitation (LOQ) of them were (*S/N* > 10, 0.03 µg/g), and LOD and LOQ of 11-oxoTTX and 4-*epi*-11-oxoTTX were 0.04 µg/g and 0.14 µg/g, respectively. MS/MS was performed in AutoMS/MS mode setting [*M* + *H*]^+^ as the precursor ions. The precursor ions and sweeping collision energy were 320.1088 ± 0.1, 36.9–55.4 eV for TTX, and 336.1038 ± 0.1, 38.1–57.1 eV for 11-oxoTTX.

### 4.7. High Resolution LC-MS for STXs

STX, dcSTX and neoSTX were analyzed using HR-LC-MS. LC was performed with a TSKgel Amide-80 column (150 x 2.0 mm i.d., particle size 5 µm, Tosoh, Tokyo, Japan) and the mobile phase, 2 mM ammonium formate in water/acetonitrile/formic acid (30:62:0.0125, *v/v*) at a flow rate of 0.2 mL/min at 25 °C in the isocratic mode with an injection volume of 2.0 µL and run time of 30 min [65]. The mass spectrometer and its condition were same as those of TTXs analysis as described above (see Section 4.4). STX, dcSTX and neoSTX were detected as [*M* + H]^+^ ions at *m/z* 300.1415 ± 0.05, 257.1356 ± 0.05, and 316.1365 ± 0.05, respectively. LOD (*S/N* > 3) and LOQ (*S/N* > 10) for STX were 0.02 µg/g and 0.07 µg/g, neoSTX, 0.05 µg/g and 0.18 µg/g, and dcSTX, 0.07 µg/g and 0.25 µg/g, respectively.

## 5. Conclusions

The concentrations of TTX and its analogues in the skin was generally higher in both *A. hispidus* and *A. nigropunctatus* both from the Solomon Islands and Okinawa, Japan, and relatively lower in the liver, ovary, testis, stomach, intestine, and flesh. Because of high concentration of TTX in the skin of these two species from the Solomon Islands (51.0 and 28.7 µg/g at highest) and Okinawa, Japan (12.7 and 255 µg/g at highest), their consumption should be banned. 11-OxoTTX, which is considered a more potent analogue than TTX by some authors, was commonly detected in the skin of all specimens of both *Arothron* species from these two regions, an occurrence that should warn citizens of a potentially higher toxicity. STX was detected in the skin of all three specimens of *A. hispidus* from Okinawa, Japan. The level of STX equivalents (STX + dcSTX) in the skin of one specimen was 3.46 µg/g, more than the global regulation level. The toxicities of other *Arothron* species from these regions should be continuously studied.
